# Supplementary material for: Genomic analysis of male puberty timing highlights shared genetic basis with hair colour and lifespan
Source: Nat Commun. 2020 Mar 24;11:1536. doi: 10.1038/s41467-020-14451-5 (PMC7093467; doi:10.1038/s41467-020-14451-5)
Supplement: Supplementary file 2 — Reporting Summary [file 41467_2020_14451_MOESM2_ESM.pdf]

nature research

Corresponding author(s): John RB Perry

Last updated by author(s): Dec 6, 2019

Reporting Summary

Nature Research wishes to improve the reproducibility of the work that we publish. This form provides structure for consistency and transparency in reporting. For further information on Nature Research policies, see Authors & Referees and the Editorial Policy Checklist .

Statistics

For all statistical analyses, confirm that the following items are present in the figure legend, table legend, main text, or Methods section.

n/a	Confirmed	
☐	☒	The exact sample size (*n*) for each experimental group/condition, given as a discrete number and unit of measurement
☐	☒	A statement on whether measurements were taken from distinct samples or whether the same sample was measured repeatedly
☐	☒	The statistical test(s) used AND whether they are one- or two-sided<br>*Only common tests should be described solely by name; describe more complex techniques in the Methods section.*
☐	☒	A description of all covariates tested
☐	☒	A description of any assumptions or corrections, such as tests of normality and adjustment for multiple comparisons
☐	☒	A full description of the statistical parameters including central tendency (e.g. means) or other basic estimates (e.g. regression coefficient) AND variation (e.g. standard deviation) or associated estimates of uncertainty (e.g. confidence intervals)
☐	☒	For null hypothesis testing, the test statistic (e.g. *F*, *t*, *r*) with confidence intervals, effect sizes, degrees of freedom and *P* value noted<br>*Give P values as exact values whenever suitable.*
☒	☐	For Bayesian analysis, information on the choice of priors and Markov chain Monte Carlo settings
☒	☐	For hierarchical and complex designs, identification of the appropriate level for tests and full reporting of outcomes
☐	☒	Estimates of effect sizes (e.g. Cohen's *d*, Pearson's *r*), indicating how they were calculated

Our web collection on statistics for biologists contains articles on many of the points above.

Software and code

Policy information about availability of computer code

Data collection	No specific software was used in data collection.
Data analysis	BOLT-LMM was used in the generation of the data relating to individual phenotype outcomes. MTAG was used for the final analysis to combine the data across the different phenotypes.

For manuscripts utilizing custom algorithms or software that are central to the research but not yet described in published literature, software must be made available to editors/reviewers. We strongly encourage code deposition in a community repository (e.g. GitHub). See the Nature Research guidelines for submitting code & software for further information.

Data

Policy information about availability of data

All manuscripts must include a data availability statement. This statement should provide the following information, where applicable:

- Accession codes, unique identifiers, or web links for publicly available datasets
- A list of figures that have associated raw data
- A description of any restrictions on data availability

Provide your data availability statement here.

Field-specific reporting

Please select the one below that is the best fit for your research. If you are not sure, read the appropriate sections before making your selection.

☒ Life sciences ☐ Behavioural & social sciences ☐ Ecological, evolutionary & environmental sciences

Life sciences study design

All studies must disclose on these points even when the disclosure is negative.

Sample size	Sample size was based on collecting the largest available sample with relevant phenotypes.
Data exclusions	We excluded people who were identified to have ancestry other than white European. This was done to remove the possibility of confounding by ancestry.
Replication	We replicated the associations in an independent sample using a range of related phenotypes.
Randomization	Not applicable
Blinding	Not applicable

Reporting for specific materials, systems and methods

We require information from authors about some types of materials, experimental systems and methods used in many studies. Here, indicate whether each material, system or method listed is relevant to your study. If you are not sure if a list item applies to your research, read the appropriate section before selecting a response.

Materials & experimental systems

n/a	Involved in the study	
☒	☐	Antibodies
☒	☐	Eukaryotic cell lines
☒	☐	Palaeontology
☒	☐	Animals and other organisms
☐	☒	Human research participants
☒	☐	Clinical data

Methods

n/a	Involved in the study	
☒	☐	ChIP-seq
☒	☐	Flow cytometry
☒	☐	MRI-based neuroimaging

Human research participants

Policy information about studies involving human research participants

Population characteristics	UK Biobank is a study of 500,000 participants aged 40 to 69, in this study only males reporting an age of voice breaking or of facial hair were used in the study.<br>The 23andMe sample comes from men aged over 18 who responded to the question 'How old were you when your voice began to crack/deepen?'.<br>ALSPAC data comes from repeated questionnaire data asked between the ages of 9 and 17 to boys in the ALSPAC cohort.
Recruitment	UK Biobank recruited 500,000 who were registered with the National Health Service (NHS) and lived within reasonable traveling distance of a total of 22 assessment centers across the UK between 2007 and 2010.<br>Data from 23andMe comes from participants of European ancestry drawn from the customer base of 23andMe.<br>ALSPAC is a birth cohort which recruited pregnant women in the Bristol area of the UK during 1990–92 and was extended to include additional children eligible using the original enrolment definition up to the age of 18 years.
Ethics oversight	Uk Biobank - UK Biobank received ethical approval from the NHS National Research Ethics Service North West (11/NW/0382).<br>23andMe - Participants provided informed consent to take part in this research under a protocol approved by Ethical and Independent Review Services, an institutional review board accredited by the Association for the Accreditation of Human Research Protection Programs.<br>ALSPAC - Ethical approval for the study was obtained from the ALSPAC Ethics and Law committee and the Local Research Ethics Committees.

Note that full information on the approval of the study protocol must also be provided in the manuscript.
