# Supplementary material for: Genomic analysis of male puberty timing highlights shared genetic basis with hair colour and lifespan
Source: Nat Commun. 2020 Mar 24;11:1536. doi: 10.1038/s41467-020-14451-5 (PMC7093467; doi:10.1038/s41467-020-14451-5)
Supplement: Supplementary file 3 — Supplementary Information [file 41467_2020_14451_MOESM3_ESM.pdf]

Supplementary information

Genomic analysis of male puberty timing highlights shared genetic basis with hair colour and lifespan

Hollis et. al.

SUPPLEMENTARY FIGURES

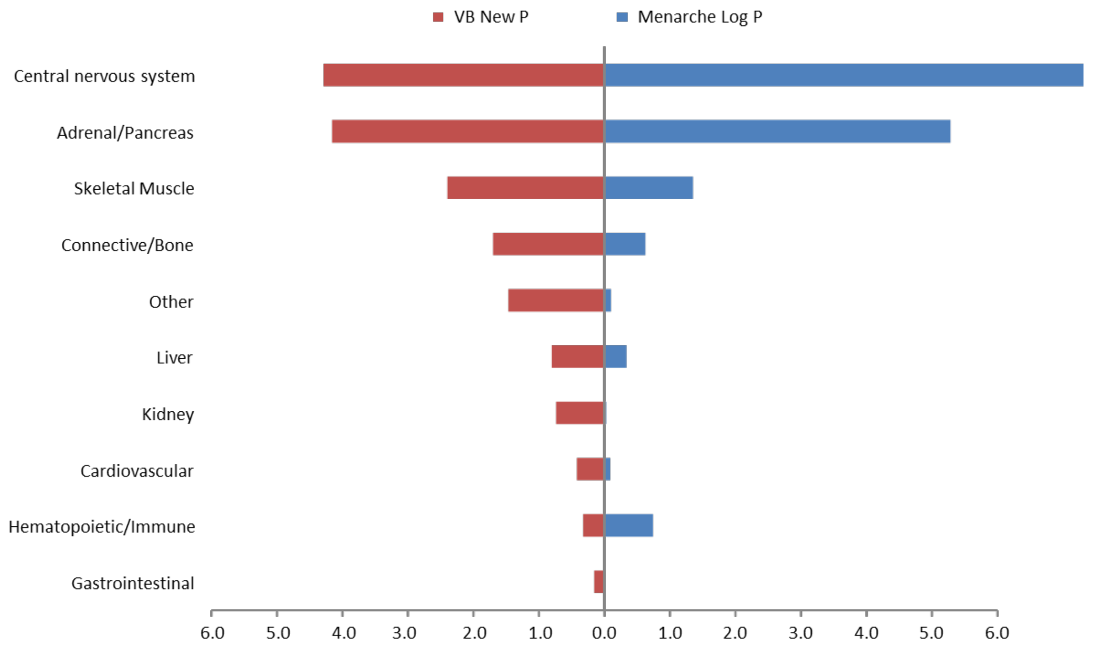

Supplementary Figure 1: Tissue expression (GTEx) for male puberty loci by general tissue categories. Values on right hand side (in red) are for voice breaking, while left is for age at menarche (in blue). Units are $-\log_{10}$ p-values (x-axis).

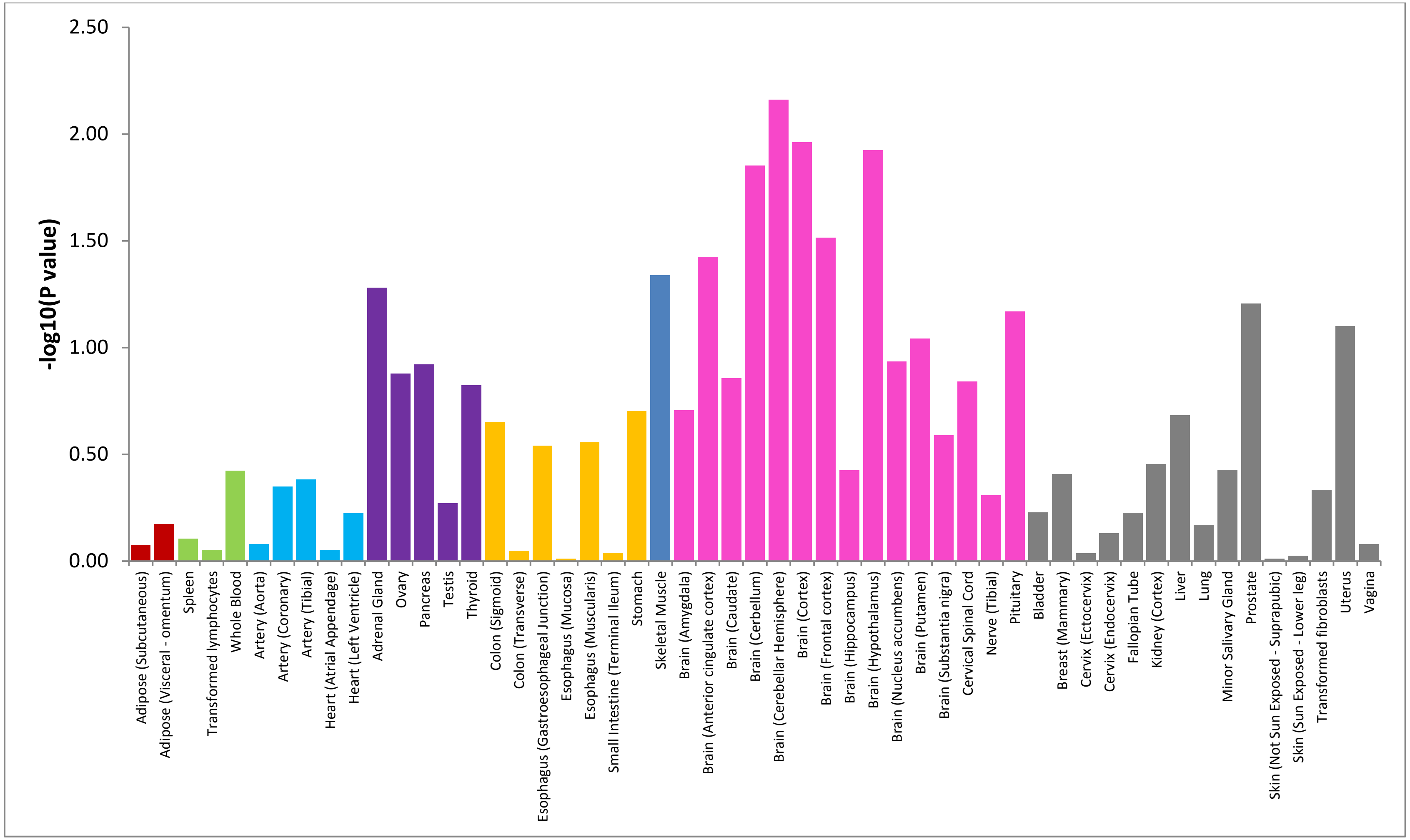

Supplementary Figure 2: Tissue expression for male puberty loci by specific GTEx tissues. $-\log_{10}$ p-values for gene expression are depicted on y-axis. Tissues are colour-coded by categories: adipose (red); blood (green); cardiovascular (light blue); endocrine (purple); gastrointestinal (yellow); skeletal muscle (dark blue); central nervous system (pink); other (grey).

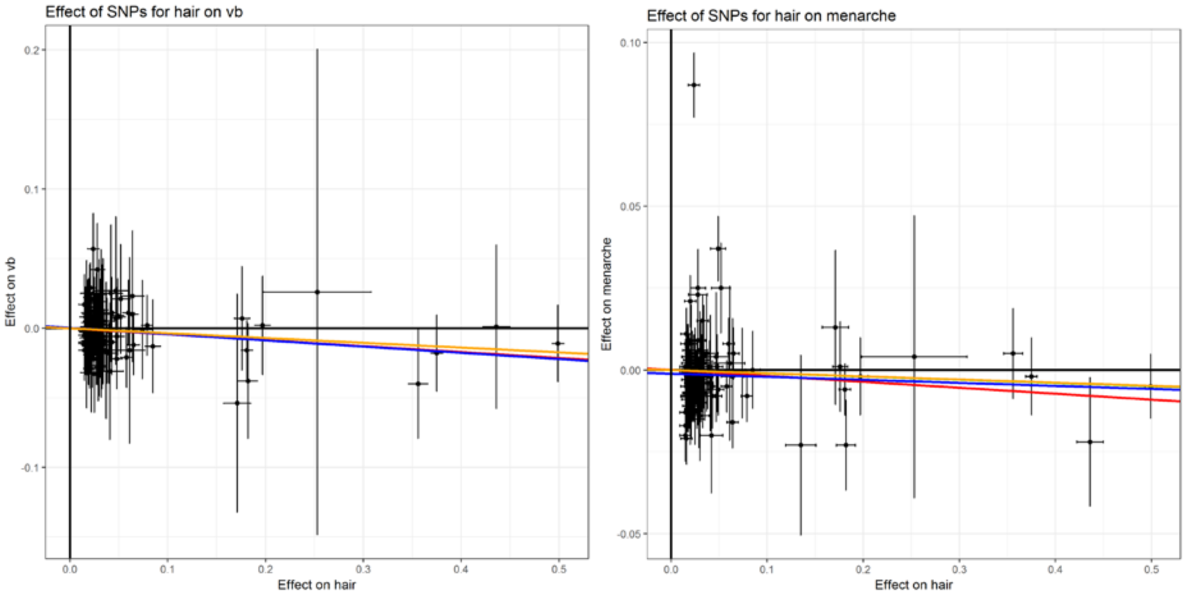

Supplementary Figure 3: Mendelian randomisation analyses for effect of hair pigmentation on puberty timing. SNP effect size on hair colour is plotted on the x-axis, while corresponding effect on age at voice breaking in men (left panel) and age at menarche in women (right panel) is plotted on the y-axis. Error bars reflect standard errors. Regression lines are shown for inverse variance-weighted (red), MR-Egger (blue) and penalised weighed median (orange) methods. Error bars represent the 95% confidence intervals for each of the SNPs plotted.

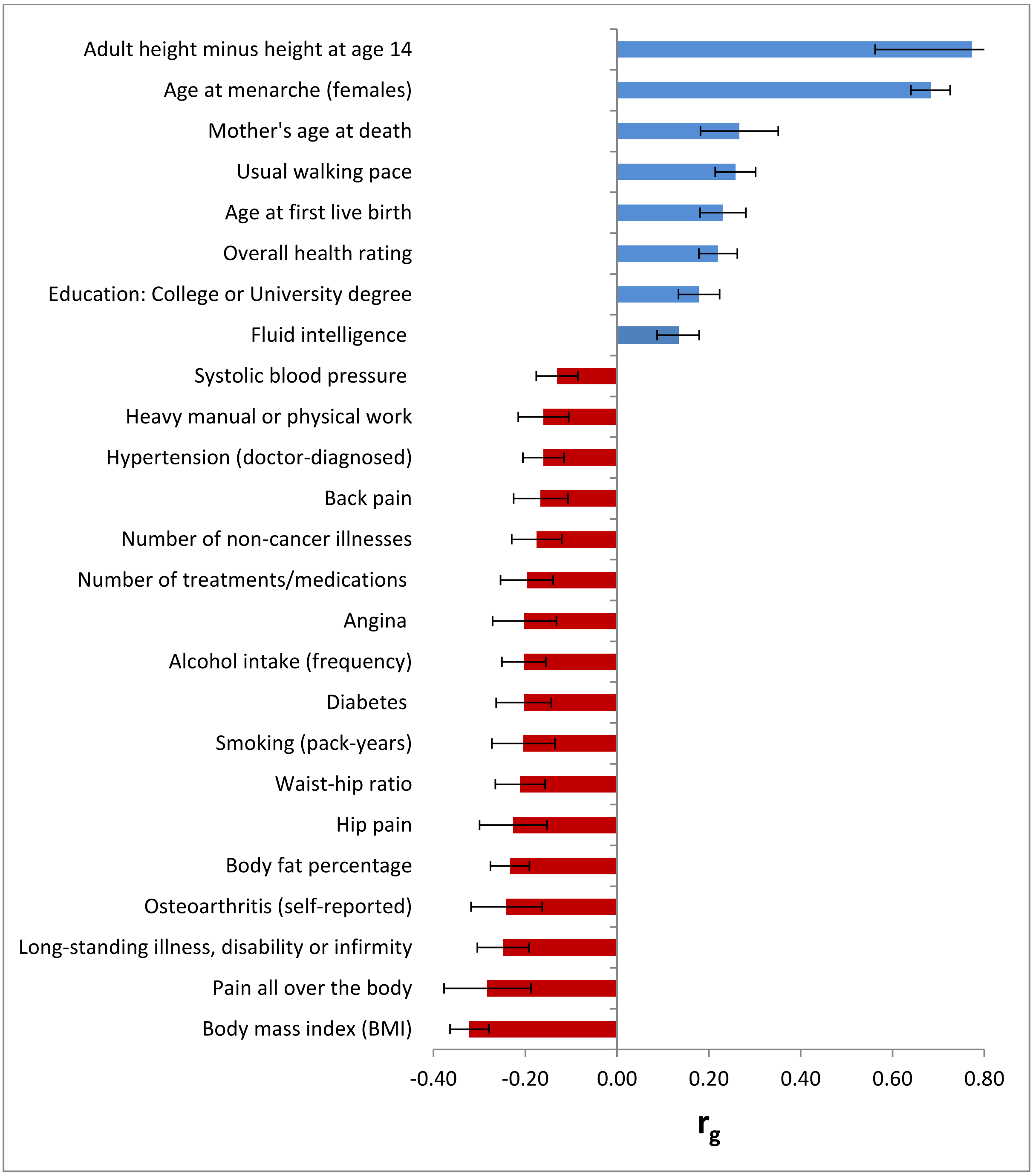

Supplementary Figure 4: Genetic correlations (r_g) between male puberty timing and selected anthropometric and health-related traits, calculated using LD Score regression. Positive correlations are shown in blue and negative correlations in red. Error bars represent 95% confidence intervals.

a)

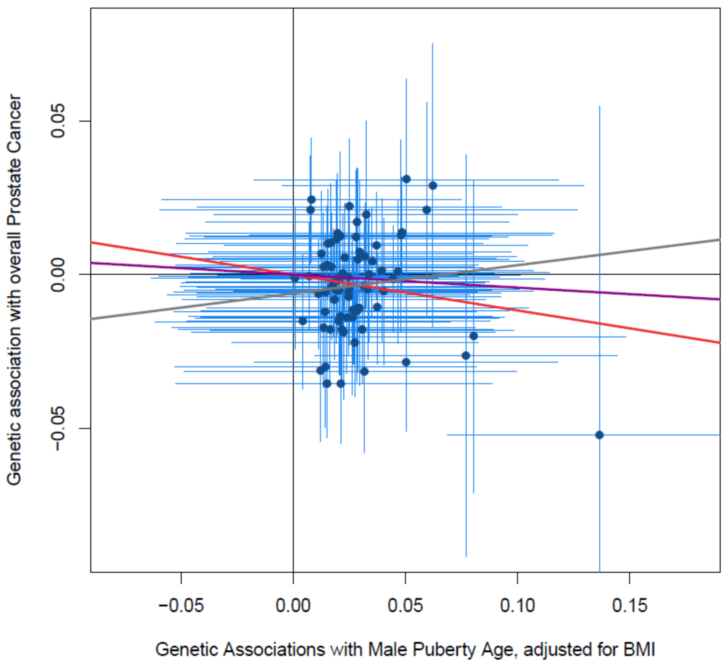

b)

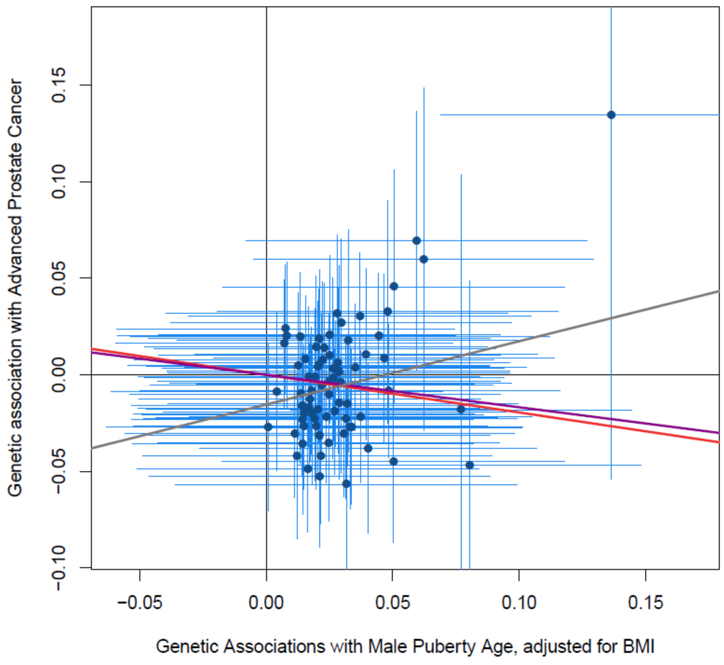

Supplementary Figure 5: Mendelian randomisation analysis for puberty timing (adjusted for BMI) to a) overall prostate cancer risk and b) risk of advanced prostate cancer. SNP effect size for puberty is plotted on the x-axis, while effect on prostate cancer is plotted on the y-axis. Blue bars represent standard errors. Regression lines for inverse variance-weighted (red), MR-Egger (grey) and weighted median (purple) methods are shown. Error bars represent the 95% confidence intervals for each of the SNPs plotted.

SUPPLIMENTARY TABLES

Supplementary Table 1 - Concordance between male puberty timing traits in UK Biobank

Facial Hair	Voice Breaking					
	Prefer not to answer	Do not know	Younger than average	About average age	Older than average	Total
Prefer not to answer	66	27	3	26	0	122
Do not know	14	4,997	74	1,375	77	6,537
Younger than average	3	892	5,952	6,254	125	13,226
About average age	23	7,037	1,980	150,303	1,832	161,175
Older than average	6	2,344	236	14,180	9,300	26,066
Total	112	15,297	8,245	172,138	11,334	207,126

Concordant %	87.1
Discordant %	12.9
Opposite % (subset of discordant)	0.2

Supplementary Table 2 - Genetic correlations between male puberty timing traits

	23andMe	UKBB Early voice break	UKBB Late voice break	UKBB Early facial hair	UKBB Late facial hair
23andMe	1	-0.835	0.808	-0.717	0.609
UKBB Early VB		1	-0.578	0.905	-0.569
UKBB Late VB			1	-0.521	0.862
UKBB Early FH				1	-0.660
UKBB Late FH					1

Supplementary Table 3 - Non-synonymous variants in LD with male puberty timing loci

GWAS SNP	Nearest Gene	Missense Variant	Missense Gene	LD (r^2)	LD (D')
rs112881196	*MEMO1*	rs9282858	*SRD5A2*	1	1
rs2186245	*LEPR*	rs1137100	*LEPR*	0.94	0.61
rs3824915	*ALX4*	rs12421995	*ALX4*	0.88	1
rs3824915	*ALX4*	rs3824915	*ALX4*	1	1
rs2049045	*BDNF*	rs6265	*BDNF*	0.96	1
rs6006984	*FAM118A*	rs11556482	*FAM118A*	0.99	1
rs6006984	*FAM118A*	rs6007594	*FAM118A*	0.86	0.98
rs438830	*CYFIP2*	rs10037485	*FNDC9*	0.98	0.99
rs767657	*HPGDS*	rs11722476	*SMARCAD1*	1	1
rs780094	*GCKR*	rs1260326	*GCKR*	0.91	0.96
rs4801593	*ZNF324B*	rs882610	*ZNF446*	0.98	1
rs4801593	*ZNF324B*	rs7257872	*ZNF584*	0.95	0.98
rs12930815	*TFAP4*	rs251732	*TFAP4*	1	1
rs60856990	*TMEM102*	rs17856697	*FGF11*	0.92	0.96
rs913588	*KDM4C*	rs913588	*KDM4C*	1	1

Supplementary Table 4 - Tissue Enrichment (GTEx) by specific tissues

Name	Category	Voice breaking		
		Coefficient	SE	P value
Adipose (Subcutaneous)	Adipose	-6.06E-09	6.12E-09	8.39E-01
Adipose (Visceral - omentum)	Adipose	-2.88E-09	6.53E-09	6.70E-01
Spleen	Blood/Immune	-5.83E-09	7.25E-09	7.89E-01
Transformed lymphocytes	Blood/Immune	-8.30E-09	6.81E-09	8.89E-01
Whole Blood	Blood/Immune	2.43E-09	7.84E-09	3.78E-01
Artery (Aorta)	Cardiovascular	-5.70E-09	5.89E-09	8.33E-01
Artery (Coronary)	Cardiovascular	7.72E-10	5.91E-09	4.48E-01
Artery (Tibial)	Cardiovascular	1.27E-09	5.97E-09	4.16E-01
Heart (Atrial Appendage)	Cardiovascular	-7.27E-09	5.94E-09	8.90E-01
Heart (Left Ventricle)	Cardiovascular	-1.62E-09	6.53E-09	5.98E-01
Adrenal Gland	Endocrine	1.12E-08	6.93E-09	5.26E-02
Ovary	Endocrine	8.46E-09	7.58E-09	1.32E-01
Pancreas	Endocrine	8.08E-09	6.88E-09	1.20E-01
Testis	Endocrine	-5.86E-10	6.50E-09	5.36E-01
Thyroid	Endocrine	6.44E-09	6.22E-09	1.50E-01
Colon (Sigmoid)	Gastrointestinal	4.96E-09	6.55E-09	2.25E-01
Colon (Transverse)	Gastrointestinal	-9.64E-09	7.67E-09	8.96E-01
Esophagus (Gastroesophageal Junction)	Gastrointestinal	3.41E-09	6.12E-09	2.89E-01
Esophagus (Mucosa)	Gastrointestinal	-1.25E-08	6.24E-09	9.78E-01
Esophagus (Muscularis)	Gastrointestinal	4.03E-09	6.89E-09	2.79E-01
Small Intestine (Terminal Ileum)	Gastrointestinal	-9.83E-09	7.05E-09	9.18E-01
Stomach	Gastrointestinal	6.31E-09	7.45E-09	1.98E-01
Skeletal Muscle	Musculoskeletal	1.25E-08	7.44E-09	4.59E-02
Brain (Amygdala)	Nervous	5.01E-09	5.88E-09	1.97E-01
Brain (Anterior cingulate cortex)	Nervous	9.60E-09	5.40E-09	3.77E-02
Brain (Caudate)	Nervous	6.12E-09	5.65E-09	1.39E-01
Brain (Cerbellum)	Nervous	1.46E-08	6.67E-09	1.41E-02
Brain (Cerebellar Hemisphere)	Nervous	1.58E-08	6.44E-09	6.94E-03
Brain (Cortex)	Nervous	1.31E-08	5.73E-09	1.10E-02
Brain (Frontal cortex)	Nervous	1.03E-08	5.53E-09	3.06E-02
Brain (Hippocampus)	Nervous	1.90E-09	6.00E-09	3.76E-01
Brain (Hypothalamus)	Nervous	1.44E-08	6.38E-09	1.19E-02
Brain (Nucleus accumbens)	Nervous	6.91E-09	5.80E-09	1.17E-01
Brain (Putamen)	Nervous	7.62E-09	5.70E-09	9.07E-02
Brain (Substantia nigra)	Nervous	3.59E-09	5.52E-09	2.58E-01
Cervical Spinal Cord	Nervous	6.54E-09	6.17E-09	1.44E-01
Nerve (Tibial)	Nervous	1.16E-10	7.16E-09	4.94E-01
Pituitary	Nervous	1.01E-08	6.77E-09	6.78E-02
Bladder	Other	-1.27E-09	5.39E-09	5.93E-01
Breast (Mammary)	Other	1.87E-09	6.83E-09	3.92E-01
Cervix (Ectocervix)	Other	-7.75E-09	5.47E-09	9.22E-01
Cervix (Endocervix)	Other	-4.28E-09	6.57E-09	7.42E-01
Fallopian Tube	Other	-1.57E-09	6.59E-09	5.94E-01
Kidney (Cortex)	Other	2.29E-09	6.05E-09	3.52E-01
Liver	Other	5.54E-09	6.81E-09	2.08E-01
Lung	Other	-3.44E-09	7.45E-09	6.78E-01
Minor Salivary Gland	Other	2.28E-09	7.06E-09	3.74E-01
Prostate	Other	1.11E-08	7.25E-09	6.25E-02
Skin (Not Sun Exposed - Suprapubic)	Other	-1.31E-08	6.48E-09	9.78E-01
Skin (Sun Exposed - Lower leg)	Other	-9.81E-09	6.08E-09	9.47E-01
Transformed fibroblasts	Other	5.60E-10	6.28E-09	4.64E-01
Uterus	Other	9.41E-09	6.69E-09	7.96E-02
Vagina	Other	-6.18E-09	6.44E-09	8.31E-01

Supplementary Table 5 - Mendelian randomisation analyses of the effect of natural hair colour on puberty timing in males and females

In males

Natural hair colour instrument (Exposure)	Age at voice breaking in years (23andMe)				
	IVW Beta:	IVW SE	IVW p-value	CochQ p-value	EGGER intercept p-value
119 SNP score	-0.044	0.016	0.0071	0.999	0.991

Natural hair colour instrument (Exposure)	Early Voice Breaking (UK Biobank)				
	Odds ratio	SE	Lower CI	Upper CI	p-value
5-SNP score controlled for 40 genetic principal components and geographical location	1.16	0.019	1.12	1.19	1.72E-19
**Natural hair colour instrument (Exposure)**	**Late Voice Breaking (UK Biobank)**				
	**Odds ratio**	**SE**	**Lower CI**	**Upper CI**	**p-value**
5-SNP score controlled for 40 genetic principal components and geographical location	0.94	0.014	0.91	0.96	5.90E-06

In females

Natural hair colour instrument (Exposure)	Age at menarche in years (ReproGen)				
	IVW Beta:	IVW SE	IVW p-value	CochQ p-value	EGGER intercept p-value
119 SNP score	-0.017	0.006	0.0036	0.038	0.472

Natural hair colour instrument (Exposure)	Age at menarche in years (UK Biobank)				
	Beta	SE	Lower CI	Upper CI	p-value
5-SNP score controlled for 40 genetic principal components and geographical location	-0.006	0.005	-0.015	0.003	0.230

Notes AAM: Age at menarche in women ReproGen consortium data (Ref. 5)

Supplementary Table 6 - Mendelian randomization analyses for the effect of puberty timing on risk of prostate cancer in men

Analysis	Odds ratio	95% CI	P-value	I^2 (%)	P_{het}	MR-Egger *P-value*
All Prostate Cancers						
Puberty timing[a]	0.94	0.84 - 1.05	0.27	48.0	2.7×10^{-6}	*0.20*
Puberty timing (adj. BMI)	0.89	0.79 - 1.01	0.06	46.4	8.2×10^{-6}	*0.1*
BMI[b]	0.90	0.81 - 1.00	0.06	49.3	4.5×10^{-8}	*0.36*
Advanced Prostate Cancer						
Puberty timing[a]	0.87	0.72 - 1.05	0.13	30.7	0.01	*0.02*
Puberty timing (adj. BMI)	0.82	0.68 - 1.00	0.05	29.3	0.01	*0.01*
BMI[b]	0.94	0.79 - 1.13	0.53	35.7	4.1×10^{-4}	*0.32*

a Risk of Prostate Cancer per 1 year later puberty

b Risk of Prostate Cancer per 1 inverse-normalised unit increase in BMI

Supplementary Table 7 - Mendelian randomization analyses for the effect of puberty timing on lifespan in men

Method	Beta[a]	SE	P-value
Inverse-variance weighted	0.09	0.03	6.7×10^{-4}
Weighted-median	0.07	0.03	0.02
Outlier-removed (MR-PRESSO)	0.09	0.03	8.3×10^{-4}

a - Beta is the self hazard protection ratio (SPHR). This is equivalent to -2×loge(Cox Hazard Ratio).
