# Supplementary material for: Genomic analysis of male puberty timing highlights shared genetic basis with hair colour and lifespan
Source: Nat Commun. 2020 Mar 24;11:1536. doi: 10.1038/s41467-020-14451-5 (PMC7093467; doi:10.1038/s41467-020-14451-5)
Supplement: Supplementary file 12 — Description of Additional Supplementary Files [file 41467_2020_14451_MOESM12_ESM.pdf]

Title: Supplementary Data 1
Description: Concordance across puberty timing trait associations for the 389 previously reported age at menarche lead SNPs

Title: Supplementary Data 2
Description: Independent genome-wide signals for male puberty timing, from MTAG

Title: Supplementary Data 3
Description: Validation of male puberty timing signals in longitudinal ALSPAC data

Title: Supplementary Data 4
Description: Heterogeneity tests for male puberty timing loci (males versus females)

Title: Supplementary Data 5
Description: Heterogeneity tests for previously reported AAM loci (females versus males)

Title: Supplementary Data 6
Description: Tissue Enrichment (GTEx) by tissue category

Title: Supplementary Data 7
Description: Results of MAGENTA analyses to identify biological pathways enriched for male puberty timing SNP associations

Title: Supplementary Data 8
Description: Genetic correlations with male puberty timing
